# Sickle Cell Disease Update: New Treatments and Challenging Nutritional Interventions

**DOI:** 10.3390/nu16020258

**Published:** 2024-01-15

**Authors:** Victoria Bell, Theodoros Varzakas, Theodora Psaltopoulou, Tito Fernandes

**Affiliations:** 1 Faculty of Pharmacy, University of Coimbra, Pólo das Ciências da Saúde, Azinhaga de Santa Comba, 3000-548 Coimbra, Portugal; victoriabell@ff.uc.pt; 2 Department of Food Science and Technology, University of the Peloponnese, 24100 Kalamata, Greece; 3 Medical School, National and Kapodistrian University of Athens, 11527 Athens, Greece; tpsaltop@med.uoa.gr; 4 CIISA, Faculty of Veterinary Medicine, University of Lisbon, 1649-004 Lisbon, Portugal

**Keywords:** sickle cell, hemoglobin, anemia, microbiota, nutrition, vaso-occlusive crisis

## Abstract

Sickle cell disease (SCD), a distinctive and often overlooked illness in the 21st century, is a congenital blood disorder characterized by considerable phenotypic diversity. It comprises a group of disorders, with sickle cell anemia (SCA) being the most prevalent and serious genotype. Although there have been some systematic reviews of global data, worldwide statistics regarding SCD prevalence, morbidity, and mortality remain scarce. In developed countries with a lower number of sickle cell patients, cutting-edge technologies have led to the development of new treatments. However, in developing settings where sickle cell disease (SCD) is more prevalent, medical management, rather than a cure, still relies on the use of hydroxyurea, blood transfusions, and analgesics. This is a disease that affects red blood cells, consequently affecting most organs in diverse manners. We discuss its etiology and the advent of new technologies, but the aim of this study is to understand the various types of nutrition-related studies involving individuals suffering from SCD, particularly in Africa. The interplay of the environment, food, gut microbiota, along with their respective genomes collectively known as the gut microbiome, and host metabolism is responsible for mediating host metabolic phenotypes and modulating gut microbiota. In addition, it serves the purpose of providing essential nutrients. Moreover, it engages in direct interactions with host homeostasis and the immune system, as well as indirect interactions via metabolites. Nutrition interventions and nutritional care are mechanisms for addressing increased nutrient expenditures and are important aspects of supportive management for patients with SCD. Underprivileged areas in Sub-Saharan Africa should be accompanied by efforts to define and promote of the nutritional aspects of SCD. Their importance is key to maintaining well-being and quality of life, especially because new technologies and products remain limited, while the use of native medicinal plant resources is acknowledged.

## 1. Introduction

Sickle cell disease, an often overlooked disease in the 21st century, is a noncontagious and enduring congenital blood disorder. It encompasses a group of clinical syndromes that affect hemoglobin due to a genetic code for abnormal polymerized deoxygenated hemoglobin. This abnormal hemoglobin distorts the shape of red blood cells, and it is inherited by children from their parents [1]. The term sickle cell disease (SCD) is derived from the polymerization of two mutant sickle β-globin subunits leading to a crescent or sickled shape of erythrocytes [2].

Sickle cell disease comprises various genotypes, yielding a group of hemoglobinopathies [3]. The production of hemoglobin is regulated by the inheritance of a pair of genes, but there is considerable variability in absolute hemoglobin levels among patients with SCD [4]. Sickle cell anemia results from the inheritance of two sickle genes, with one gene from each parent [5,6].

Two parts, heme and globin, constitute the normal form of hemoglobin. The protein is made up of four polypeptide chains (two α chains and two β chains). There are many known mutations in the hemoglobin subunit β-HBB (β-globin protein) coding gene, which make up the most common form of hemoglobin in adult humans, hemoglobin A (HbA) [7]. A variety of inherited diseases arise from these mutations. Abnormal versions of β-globin, such as hemoglobin C (HbC), hemoglobin E (HbE), and hemoglobin S (HbS), are produced by a variant mutation in the HBB gene. It is this mutation in the HBB gene that causes sickle cell anemia [8]. 

Sickle cell anemia (SCA) is the most prevalent and serious genotype of SCD, followed by HbSC (“mild” form of SCA), hemoglobin (Hb) Sβ thalassemia, HbSβ+thalassemia (accounting for some 30–40% of SCD patients), and other rare and benign genotypes [9,10]. 

Sickled red blood cells are susceptible to chronic hemolysis [11], and emerging evidence reveals that SCD is made evident by the presence of chronic inflammation and oxidative stress, both of which play a role in the development of chronic vasculopathy and several other enduring complications [12]. SCA, characterized by abnormal red blood cells and hemoglobin, is worsened by low oxygen levels in the air [13].

**Figure 1:**
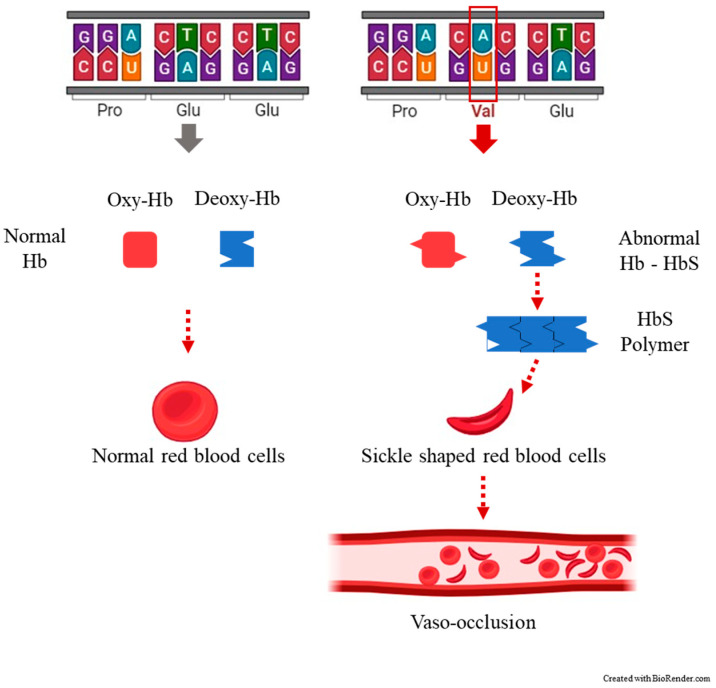
The most common clinical manifestation of sickle cell disease, a vaso-occlusive crisis (VOC) occurring when blood flow is blocked by sickled red blood cells (crescent-shaped) to the point that tissues and organs become deprived of oxygen, causing pain.

SCA is manifested as the result of the presence of an autosomal recessive allele, which is found on the short arm of chromosome 11p15.5 [14]. This alteration of the genetic code leads to the substitution of a single amino acid, where valine replaces glutamic amino acid in the sixth position of the 146 amino acids of the β chain of hemoglobin [5] (Figure 1).

After more than one hundred years since the discovery of sickle cell group of hemoglobinopathies as genetically inherited diseases [15], new studies are still necessary to explore the molecular mechanisms leading to fetal hemoglobin induction and find ways to reduce the adverse effects in patients with SCA and other β-hemoglobinopathies [16]. 

There are both severe and mild SCD genotypes, which reflect the type of symptoms and prognoses for the disease. Research has been conducted during routine patient care to identify possible clinical biomarkers among SCD patients. These biomarkers may vary according to genotype and treatment categories. However, there is still insufficient progress in developing treatment options or counseling decisions [17,18,19].

Sickle-shaped red blood cells are more rigid and stickier, which leads to the obstruction of small blood vessels. This obstruction prevents oxygen from reaching body tissues and organs, inducing both acute and chronic intense pain. There is little research focusing on the pathophysiology of acute or chronic pain in SCD, and therefore it is still poorly understood. However, it is believed to be dependent on the interaction of several molecular mechanisms [2,20,21].

### 1.1. The Incidence of Sickle Cell Disease

This disease substantially induces multimorbidity and impairs quality of life, while placing strain on healthcare systems wherever it exists [22,23]. The global burden of this disease has been assessed [24], highlighting the high risk of child mortality associated with SCA. In Sub-Saharan Africa, it can contribute to as much as 90% of under-5 mortality [25,26], with approximately 500 children with SCD continuing to die prematurely every day [27]. This is due to delayed diagnosis and/or the lack of access to comprehensive care, a trend that urgently needs to be reversed [9]. 

Every year, between 300,000 and 400,000 newborns with SCA are delivered around the world, whereas tens of thousands of people show the homozygosity for hemoglobin S form, which represents the most severe clinical phenotype of the disease [28]. Although SCD occurs worldwide, Sub-Saharan Africa is the region with the highest prevalence. It is estimated that approximately 1000 children with SCD are born in Africa every day, and more than 500 of them die before reaching the age of 5 years [29].

Children suffer several preventable chronic disorders that are followed by premature death associated with SCD. Efforts have been made to identify achievable goals to improve outcomes both in the short and long term. These initiatives aim to recitfy the present unfair attention given to this inherited condition, particularly in developing countries [30]. 

Approximately 1 in 12 African Americans carries the SCD mutation, and 1 in 500 African Americans suffers from the disorder. In the U.S., 1 out of every 16,300 Hispanic-American neonates is born with SCA each year [31]. Epidemiological data on all blood disorders is still scarce, but SCD is estimated to affect approximately 250 million people globally. 

Despite the increase in the global burden of SCD, which is believed to affect over 20 million people [32], including an estimated 200,000 annual sickle genotype births in Sub-Saharan Africa [33], available data on SCA prevalence, morbidity, and mortality remains limited on a global scale. However, some systematic reviews on global data exist [34,35].

Different areas in Côte d’Ivoire, Egypt, Lake Chad, Sudan, Lake Victoria, the coast of Kenya, Tanzania, Mozambique, and the east coast of Madagascar have been projected to have a a predicted HbS allele frequency between 7.5% and 12.5% [36]. In northern Mozambique, hematological studies have revealed a prevalence of sickle cell trait (HbAS) and G6PD (glucose-6-phosphate dehydrogenase) deficiency to be around 4% [37,38].

Sickle cell trait (HbAS) is notably more common in West Africa. It is very interesting and well-known that carriers of the sickle cell trait HbAS experience natural and nearly complete protection against severe *Plasmodium falciparum* malaria. This protection is observed despite the inadequately understood relationships between HbAS, malaria, and other common causes of child mortality [39,40,41,42].

### 1.2. Sickle Cell Disease Physiopathology

When cells are subjected to physiological stressors, they react with a mechanism described as the heat shock response. This mechanism activates a certain type of critical molecular regulator called heat shock proteins (HSPs) [43]. 

Heme oxygenase 1 is a member of the heat shock protein (HSP32) family and is involved in numerous cellular operations [44]. Increased heme in SCD causes the upregulation of heme oxygenase 1, which leads to cardiomyopathy through ferroptosis, an iron-dependent nonapoptotic form of cell death [45].

This enhances the fact that both genetic and environmental factors affect the process. Thus, the understanding of biomarkers and the molecular basis of diseases such as SCA are significant in playing a definitive role on the onset of such pathologies and, therefore, on the prevention strategies [46,47].

**Figure 2:**
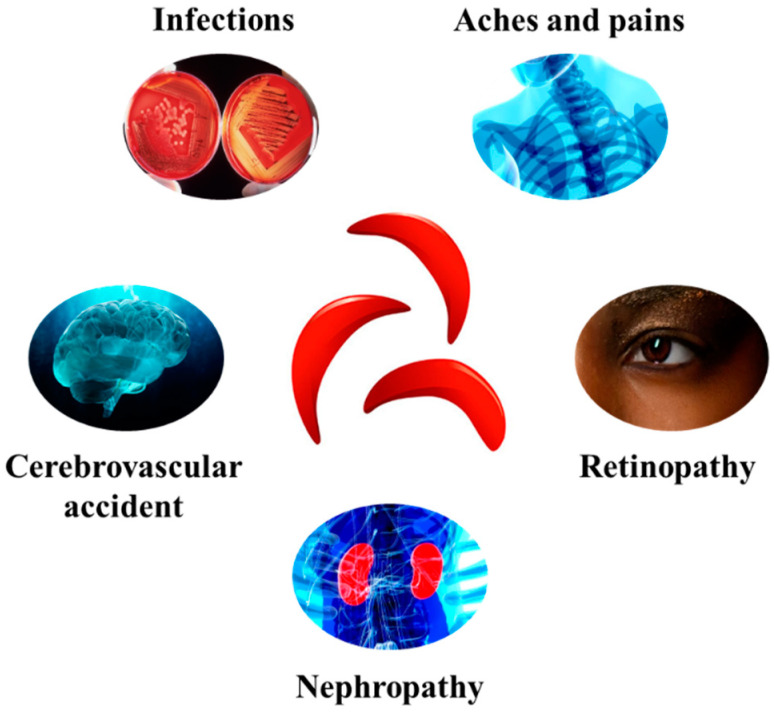
Some of the major complications associated with SCD development.

This leads to the formation of hemoglobin S and the change to sickle-shaped red blood cells compared to normal red blood cells. These cells obstruct the bloodstream, hence leading to serious problems, including cerebrovascular accident, nephropathy, retinopathy, infections, aches, and pains [48] (Figure 2).

Among the four DNA bases (two purines: adenine and guanine, two pyrimidines: thymine and cytosine), guanine has the lowest redox potential and is preferentially targeted for oxidation [49]. Despite guanine reduced redox potential, guanine radicals are known to trigger mutations and damage the genetic code, which are involved in carcinogenesis and ageing [50].

Because SCD is hallmarked by an underlying chronic inflammatory status, which is partly driven by proinflammatory M1 macrophages [51], heme scavenging or modulation, as well as the potential therapeutic targeting of mitochondrial biogenesis, might significantly ameliorate tissue damage associated with SCD pathophysiology [52].

Peroxisome proliferator-activated receptor-γ coactivator 1-α (PGC1α), a transcriptional coactivator protein that regulates the genes involved in energy metabolism [53], exerts significant control over, induces, and coordinates gene expression. It stimulates mitochondrial oxidative metabolism (i.e. respiratory capacity, oxidative phosphorylation, and fatty acid β-oxidation), produces ATP and lipids, induces amino acid and heme biosynthesis, and generates/sequesters reactive oxygen species (ROS) [54].

### 1.3. Sickle Cell Disease Diagnosis

Sickle cell disease can be prevented prenatally, and it can also be diagnosed in utero or in the newborn period through screening. Early diagnosis of this condition is essential for beginning treatments that can reduce the risk of life-threatening complications, such as severe infections and strokes, as well as managing the disease effectively to reduce morbidity. SCD is diagnosed through a simple complete blood test, peripheral blood smears, hemoglobin electrophoresis, HPLC, and various genetic sickling tests. Hemoglobin S solubility assay and sodium metabisulfite test may be used for screening individuals aged 6 months or older. For pregnant women, screening should ideally be conducted before 10 weeks’ gestation. Recent studies have also reviewed current emerging portable techniques that have been developed for the early detection and diagnosis of sickle cell disease and carrier states [55,56,57]. More detailed molecular genetic diagnose testing is also available [58].

## 2. The Advent of New Technologies

The discovery of CRISPR (clustered regularly interspaced short palindromic repeats) and its function, which took place between 1993 and 2005, marked the recognition of acquired immunity systems that are widespread in archaea and bacteria. This discovery has since been widely validated through the development of CRISPR and CRISPR-associated protein (Cas) systems [59]. As a result of the emergence of genome editing tools, this subject has experience significant growth, with universities, research institutes, biotechnology enterprises, and sizable pharmaceutical companies collaborating to create innovative therapeutics with far-reaching potential [60,61].

Using CRISPR-Cas technology, which is a specific, efficient, and versatile gene editing technology, one can modify, delete, or correct precise regions of DNA [62,63]. However, this gene therapy still needs to be economical, practical, easy, explicit, quick, convenient, safe, and adequately valid to be capable of producing the desired effect [64]. 

Formerly, gene editing required tissue samples to be removed from the body for editing outside, but now it is possible to use this technology in vivo. Thus, genome editing therapies can be developed to silence “bad” genes [65,66,67].

CRISPR-Cas9, which depends on ribonucleoprotein complexes (RNPs), leverages the subcellular location of mRNAs transported within cells in RNPs and is indeed a powerful tool for targeting and editing DNA [62,68,69].

Since the early 2000s when the identity and clinical functions of microRNAs (miRNAs) [70] were discovered, the roles of miRNAs as potential biomarkers for both diagnosis and prognosis have been actively investigated over the past few decades [6,71,72].

MicroRNAs, which are short noncoding genetic material implicated in the modulation of mitochondrial activity and homeostasis, also contribute to the readjustment of cell metabolism, offering a new perspective on the regulation of gene expression following transcription [73]. They act by enhancing the activity of apoptosis-inducing factors and by targeting and eventually silencing specific genes. This silencing process involves known oncogenes and disease suppressor genes related to metabolic signaling pathways and is associated with genetic disorders [73,74].

Patients with high fetal hemoglobin (HbF) status, which is a product of γ-globin genes and modulates SCD, experience fewer painful crises and enhanced survival rates [75]. Examining the factors that control γ-globin genes at both transcriptional and translational levels, including miRNAs, can assist in the identification of possible therapeutic avenues for SCD [76,77].

Some miRNAs have the potential to serve as valuable molecular tools for innovative therapeutic approaches in hemoglobinopathies, especially in the context of hematogenesis, erythrocyte cell differentiation, and degree of anemia severity. Using these miRNAs could ameliorate the clinical framework of SCA [78]. 

Patients with SCD often display abnormal triggering of the innate component of the immune system’s natural defense pathway, leading to higher risks of infection and predisposing patients to autoimmune diseases [79]. 

The future of medical research will be to focus on the identification and development of noninvasive biomarkers for specific diseases. However, it is still uncertain which miRNAs, or combinations of multiple biomarkers, could be the most prominent candidates for discovery and development [80]. 

## 3. Current Treatments of Sickle Cell Disease

A definitive cure for sickle cell anemia (SCA) remains a subject of debate. In this review, we only aim to summarize current trends and knowledge regarding available treatments for SCD and to emphasize the fact that there is a substantial unmet need for medicines. We will also provide a brief overview on existing therapeutic interventions worldwide, which are largely limited to blood transfusions. 

Increasing the production of fetal hemoglobin (HbF) in significant quantities can diminish the severity of the clinical progression in β-thalassemia and SCD. This can lead to a decrease in morbidity, disability, impairment, illness, and mortality [81]. 

There are science-based guidelines elaborated by the American Society of Hematology (ASH) designed to support patients, clinicians, and other healthcare professionals, namely, in pain management decisions for children and adults with SCA. However, these do not provide specific guidance on nutritional care and other strategies [82,83].

SCD is caused by a mutation that results in the substitution of glutamic acid for valine. Despite many gaps in our understanding of the biological mechanisms of glutamine and its therapeutic implications, the FDA approved L-glutamine (10–30 g/day oral powder, twice daily) in 2017 for individuals aged 5 and older to lower the number of pain crises [84]. 

Until recently, only the use of an oral chemotherapeutic drug, hydroxycarbamide (also known as hydroxyurea), was considered for the treatment of SCD. Hydroxyurea, which is a ribonucleotide reductase, is the only approved drug for disease-modifying treatment in patients with SCA [85]. However, it is currently underutilized in clinical practice [86].

A class of new medications called hemoglobin S (HbS) polymerization inhibitors (e.g., voxelotor), has been recently approved by the FDA in 2019 and by the E.U. EMA in 2022. These drugs are intended for the oral treatment of hemolytic anemia due to SCD and vaso-occlusive crisis (VOC), in adults and children aged 12 years and older [87].

This small-molecule drug is able to attach to and stabilize hemoglobin, preventing hemoglobin polymerization (i.e., formation of abnormal hemoglobin) that causes the formation of sickle shaped red blood cells [88]. In well-resourced countries, three potential treatments are available for preventing or reducing the morbidity and mortality associated with SCA: transfusions, hydroxyurea, and stem cell transplantation [89]. There is no evidence of any benefits of corticosteroid use in SCD acute events [90].

The polymerization of abnormal hemoglobin S upon deoxygenation in the tissues to form fibers in red cells causes the development of SCD, thus, generating deformations and blockages in the circulation. Hence, many attempts have been made to find drugs that can control nonpolymerizing fetal hemoglobin [88].

Vaso-occlusive crisis has been prevented and treated using an approved drug called crizanlizumab. This drug is designed to treat pain by preventing blood cells from sticking to the inner walls of blood vessels. The monthly administration of this monoclonal antibody against P-selectin (mediator of inflammation through promoting adherence of leukocytes to activated platelets and endothelium) has proven effective in lowering the frequency of sickle pain crises [91].

There is a hypothesis, which requires further investigation, suggesting that leucine transcriptional nuclear factor NRF2 activation with sulforaphane (a chemical compound found in vegetables such as broccoli and Brussels sprouts), may offer therapeutic benefits for SCD patients. These potential benefits could include reducing liver damage, restoring oxidative capacity, and increasing fetal hemoglobin concentration [92]. 

Allogeneic hematopoietic stem cell transplantation, also referred to as bone marrow transplant, has been known to cure severe congenital anemias. This treatment has been used to transplant healthy hematopoietic stem cells, obtained from several sources, into patients with dysfunctions related to many malignant and nonmalignant disorders [93] (Table 1).

### Recent Advanced Therapeutic Approaches for Sickle Cell Disease

Currently, the sole validated healing medicinal approach for SCD is allogeneic (genetically dissimilar) hematopoietic stem cell transplantation, ideally from an unaffected human leucocyte antigen (HLA)-identical matching sibling donor, when available. Combining genetic treatments and hydroxyurea seems to provide the best results [103]. However, normally, this is only considered for children younger than 16 who have severe complications from the disease [104,105,106,107].

Although still under review in the U.S., the European Union, and Saudi Arabia, the Medicines and Healthcare products Regulatory Agency (MHRA) in the U.K. has conditionally approved an innovative and first-of-its-kind gene editing therapy for patients aged 12 and over with SCD and transfusion-dependent beta thalassemia. It is worth noting that in the U.K., there are approximately 15,000 SCD patients.

These treatments, known as Casgevy^TM^ and Lyfgenia^TM^, which have been approved by the FDA, are designed to work by editing the faulty gene in a patient’s bone marrow stem cells. They are the first medicines to be licensed using the innovative gene editing tool CRISPR. The inventors of Lyfgenia^TM^ were awarded the Nobel Prize in 2020 [102]. Following the U.K.’s authorization to use Casgevy^TM^, Bahrain was the second country to approve the use of the drug, and in December 2023, the U.S. became the third.

A drug called masitinib, which has been available in Europe since 2008 for veterinary use under the name “masivet”, a tyrosine-kinase inhibitor used in canine cancer treatment, is presently being tested for the treatment of SCD. A new clinical development program for a phase two clinical trial of masitinib in SCD has been granted a substantial investment to study the involvement of mast cells and basophils in orchestrating acute and chronic complications of SCD. A new patent has been filed, which, if granted, will extend the international protection of masitinib for use in treating SCD until 2040 [108]. 

Microglia from the brain and spinal cord and mast cells (bone marrow-derived tissue-dwelling cells), two key immune cells involved in numerous pathologies, release substances such as histamine, leukotrienes, and cytokines, causing inflammation. This process is strongly related to SCD [109]. 

## 4. Influence of Gut Microbiome in Sickle Cell Disease

The gut microbiota, their genes (microbiome), and metabolites express a large number of potential signaling molecule ligands and metabolites. These components, under homeostatic conditions, can control inflammatory interleukins, growth factors, cytokines, and prostaglandins in coordination with tissue immunity [110,111].

The gut microbiota, which accommodate approximately 10^13^–10^14^ cells belonging to a diverse group of microorganisms [112] (equivalent to over 3 million genes), play a key role in human health by determining the development of conditions linked with the nervous system, autoimmunity, metabolism, and inheritance [113,114].

Increased gut injury and permeability, modified microbiota composition, and bacterial overgrowth, along with collateral bacterial translocation in SCA patients, reveals a state of dysbiosis and an increase in gut microbiota responsible for triggering inflammation [115]. 

Gut microbiota are closely involved in energy homeostasis [116], immune system regulation [117], metabolism [118], and other physiological processes of hosts, as reported by research studies. 

A disruption of the microbiome spawns considerable dysbiosis. This dysbiosis is observed in SCD patients because some bacteria trigger proinflammatory responses and can affect some of the pathophysiological features of this disease [119]. This microbial imbalance was mainly recorded in the U.S., as SCD patients often are hospitalized and subject to nosocomial infections. However, in Africa, where SCD prevails, not many studies have been conducted, and the gut microbiome profile is associated with race and/or ethnicity [120]. 

A complex interplay occurs between environmental (e.g., diet and medications) and host factors within the gastrointestinal tract, where symbiotic microorganisms reside. This interplay is affected by the human genome, which affects the gut microbiome through enzymes and miRNAs [121]. The target for customized nutrition and therapy, as well as the renovation of exchanges between the microbiota and host, and the modification of nutrition to restore the necessary symbiosis, is microbiota modulation. The aim is to reverse established microbial dysbiosis [122,123,124].

A crucial mediator in the immunomodulation of inflammation, cell adhesion, and induction of aged neutrophils, which are the main arbitrators of recurrent VOC, is diverse gut microbiota [125]. 

Several studies have shown that host genetics affect the composition or structure of the gut microbiome and vice versa [126,127]. However, our understanding of the association between host genetics and the gut microbiome in complex human diseases remains limited. It is still challenging to estimate the scale to which host genetics shape the configuration of the gut microbiota [128,129].

To understand the complex pathophysiology of the disease and the evaluation of potential specific therapies, researchers have developed several murine models and generation of transgenic mouse clones as a solution for the absence of a natural animal pattern for SCD [130,131,132]. Most studies on this interaction have therefore been conducted using genetically engineered mouse models. Results from these studies have strengthened our knowledge of host genetics and its influence on the human gut microbial variation despite the existing differences between the microbial composition of humans and mice [133,134].

Therapeutic strategies involving synergistic gene addition and gene silencing in stem cell progeny have demonstrated proof of concept through targeted research [135]. The gut microbiome has been characterized in murine models with SCA, revealing significant dysbiosis [115]. The gut microbiota is believed to play a role in the severity of SCD because the permeability of the intestinal barrier is compromised. This is connected with gene silencing of continuous intercellular network proteins, enhanced inflammation, and oxidative stress, which are all specific to the small intestine [136].

The generation of ROS and other free radicals occurs during normal cellular metabolism, and inflammation and oxidative stress are intertwined, one aggravating the other. The therapeutic potential of natural food antioxidants has been widely researched, namely, in chronic metabolic disorders [137,138].

SCD patients show decreased protection from antioxidants in their blood, possibly due to lipid peroxidation, which results from its interaction with ferroptosis and a compromised antioxidant competence [139]. When administered at appropriate doses, omega-3 fatty acids (ω-3 FAs), specifically docosahexaenoic (DHA) and eicosapentaenoic (EPA) acids, are potent anti-inflammatory mediators that modulate pain. They also decrease episodes of VOC in SCD [140]. The therapeutic effects of polyunsaturated ω-3 fatty acids in SCD have been shown in clinical trials, providing increasing evidence for a safe and effective treatment for SCD, demonstrating improvements in VOC rates, biomarkers of inflammation, cell adhesion, and hemolysis [141,142].

Chronic sickle cell pain and osteoporosis are common clinical manifestations in humans, but their underlying causes are not fully understood. It appears that the gut microbiome may play a role in the management of chronic SCD pain. Increased gut tissue injury and permeability and bacterial translocation of luminal contents contribute to the role of the microbiome in SCD [115,143].

It is also suggested that gut dysbiosis in SCD induces pain through changes in vagal nerve activity [144]. Increased inflammatory cytokines, which are critical mediators that oversee and regulate immune and inflammatory responses via complex networks serving as biomarkers, arise from increased gut bacteria burden and augment antigenic load, travelling across the impaired intestinal barrier through inflammation [145] (Table 2). 

The identification of therapeutic approaches for gut modulation is still in its early stages despite the evaluation of the microbial differences between SCD patients and healthy controls in some studies [26,146].

## 5. Nutritional Perspectives in Sickle Cell Disease

Nutritional imbalances are considered as crucial factors contributing to the severity of sickle cell disease. This has led to increased interest in promoting dietary supplementation for treating patients, especially because no cure for sickle cell anemia is available. Patients with sickle cell disease require higher energy and protein intake compared to healthy individuals. These patients tend to suffer from undernutrition if their energy intake is consistently low [147].

There has been little researched on dietary interventions that may be useful as a supplementary tool to treating SCD. However, it is well-established that unbalanced nutrition is a significant risk factor that adversely impacts clinical events, welfare, vital processes, and the independence of patients [148].

There is a deficiency in knowledge and the possible integration of nutrition into sickle cell medial services. Awareness must be raised regarding the value and importance of the role of nutrition in improving the management and care of SCD, particularly in Africa [149]. Nevertheless, helpful dietary suggestions, especially for children with SCD, must be a priority.

Major factors contributing to the increased severity of SCD include chronic inflammation and oxidant stress. Hence, the establishment of recommended reference intakes for SCA patients is fundamental, and nutritional intervention should be included as supplementary care in conjunction with standard practices [150].

Because no easy and cost-effective treatment has yet been found, recent efforts have been directed toward nutritional interventions to reduce ill health and improve the quality of life for SCD patients. However, there are limitations within the current scope of the recommended daily allowances and dietary reference intakes for world populations, and nutrient density has also been under consideration [151]. This makes it even more challenging to establish specific nutritional requirements for sickle cell patients.

The role of malnutrition as one of the complications of SCA and the possible benefit of regular micronutrient supplements have been recently demonstrated by several authors [152,153]. Symptoms occur around the age of 5 months, but they vary among individuals and are characterized by episodes of pain, fatigue, frequent infections, organ damage, and early mortality. These symptoms can cause delayed growth and development in children while resulting in a need for a higher amount of certain nutrients, including calories and protein [154].

Due to chronic ischemia-reoxygenation damage induced by VOC, patients with SCD may suffer from leaky gut, which affects microbiota density and adhesion to the epithelial wall and the degree of translocation [155]. This influences nutrient intake, metabolic homeostasis, hormonal environment, microbiota imbalance, and immunity [156]. 

Particular attention has been directed to identifying dietary deficiencies that often coexist with SCD and searching for novel dietary strategies to reduce morbidity and improve the quality of life for these patients [157,158]. SCD has been also associated with vitamin D deficiency and poor appetite. Although a few studies have been conducted, none have been of sufficient quality to guide clinical practice [159]. 

Recognizing gene–nutrient interactions with the objective of explaining a specific response in various ethnic and environmental contexts is a complex task [160]. The prevalence of nutritional deficiencies in SCD is evident, and they may be associated with worse pain outcomes [119]. Nutrition, being a complex and wide-ranging issue, calls for the implementation of a new collaborative approach that addresses staple food types and sources, micronutrients, and phytonutrients, which are all critical for optimal human health and well-being. Particular focus should be given to improved digestibility, the human gut microbiome, overall vitality, and mental health [161,162].

Children with hereditary diseases such as SCD frequently have feeding disorders and dysphagia as a result of the elaborate interplay between anatomical, physiological, medical, and behavioral factors. These feeding complications may also cause food consumption to be troublesome, passive, or even painful because of a lack of breath and ability to talk, choking, coughing, tiredness, or vomiting, causing the child to stop eating and requiring a parent to feed their child.

Dietary intake has been inadequately considered in SCA, and questionable guidance for dietary iron restriction has been assumed, although iron deficiency is unexpected in SCD patients based on the fact that the homozygous SCA genotype is associated with the most severe form [163].

It has been proven that the disease mechanism of SCD has considerable nutritional and health connotations, including elevated energy and nutrient requirements, nutrient deficiencies, and growth abnormalities [164,165]. However, these data were obtained using small sample sizes, namely in Sub-Saharan Africa, where it prevails. Hence, the need for further investigation of the potential benefits of nutrition-related interventions for these patients is imperative [166]. Over the last decade, the prime concerns for basic, clinical, and demographic research regarding food, diet, supplements, and nutrition in individuals with SCD and thalassemia have been suggested as main areas of research and innovation [167,168].

The main obstacle has been difficulty in the assessment of dietary intake, nutritional status, the use of nutritive and nonnutritive dietary supplements, and increased vulnerability to infections caused by specific pathogens in these patients, particularly children under 5 years of age [169]. 

The risk of a worse prognosis in SCA has increased due to inadequate food and nutrient intake data in developing countries [170]. Plans to motivate the intake of minimally processed foods should be considered due to the benefits of antioxidants acting positively against SCA [171,172].

Micronutrient deficiencies in these patients may increase vulnerability to stunting, inflammation, opportunistic infection, and acute painful crisis [173]. A number of micronutrient deficiencies and their associations, including iron, zinc, copper, folic acid, pyridoxine, and vitamin E, have been long considered and addressed [174]. While it is possible that folic acid supplementation may increase serum folate levels, the effect of supplementation on SCA remains unclear [175]. 

Randomized clinical trials have assessed the efficacy of antioxidant nutrient supplementation in reducing hemolysis in SCD patients. It found that vitamin C and E, when used in safe doses, were considered to worsen hemolysis [176]. However, supplementation with ω-3 fatty acids, vitamin A, and zinc were reported to improve indirect hemolytic markers [172].

Some preliminary meta-analyses have found a that supplementation with the semi-essential amino acid L-arginine or its precursors has a beneficial effect on patients with SCD. The conversion of proline, glutamate/glutamine, and the nonproteinogenic amino acid citrulline leads to the endogenous synthesis of L-arginine [177,178], which plays an important role in cell division, the healing of wounds, the stimulation of protein synthesis, immune function, and the release of hormones [179]. 

Morbid occurrences in SCA lead to an increase in the generation of reactive oxygen species (ROS) through the activation of several prooxidant enzymes. Under normal conditions, these enzymes would be balanced between oxidant and antioxidant systems to prevent oxidative damage. However, the injury of sickle red blood cells causes hemolysis, the release of free hemoglobin, the modification of mitochondrial respiratory chain activity, and red blood cell autooxidation [180]. 

A surplus of free radicals can damage cells, causing illness and aging and contributing to greater oxidative stress in erythrocytes, endothelial cells between the bloodstream and the surrounding tissues cells, polymorphonuclear leukocytes, and thrombocytes. This oxidative stress is associated with multiorgan disorders, vasculopathy, and cellular dysfunction [181]. 

Despite the capacity, constraints, and frustrations of antioxidant treatment to date, many foods containing powerful antioxidant enzymes may play an essential role in contributing to the mitigation of oxidative-related obstacles. This enables the development of nutritional strategies aimed at improving antioxidant status in SCA patients [182]. 

However, free radicals react too quickly with lipids, proteins, and nucleic acids in cell membranes, making it challenging for exogenous monomers to scavenge them effectively [183]. Hence, the idea of collecting these radicals in biological systems using exogenous compounds has been considered impractical [139]. 

It is not the deficit of essential polyunsaturated fatty acids in the diet, but rather the modified metabolic pathways of fatty acid elongation and desaturation on the endoplasmic reticulum membrane in children with SCD that leads to reduced polyunsaturated fatty acids in the phospholipids of the cell membranes, which contributes to known disease symptoms [184].

The possible nutritional strategy, when used as a complement to adequate treatment, must focus on mediating the mechanism of a free-radical substitution reactions. This involves maintaining a balance between the formation and removal of free radicals, which can be achieved through a diet rich in healthy, high-antioxidant foods [185]. 

In individuals with SCD, the antioxidant defense system is greatly compromised due to depleted expression and activity levels of antioxidant enzymes (e.g., superoxide dismutase-SOD, catalase-Cat, and glutathione peroxidase-GPx). These enzymes play a crucial role in breaking down hydrogen peroxide and thus controlling its intracellular concentration [186,187].

### 5.1. African Plant Resources in Sickle Cell Disease

There is the need in Africa to embrace both natural and pharmaceutical medicine, creating a harmony between nature and science and dismissing any stigma associated with sickle cell disease. The exploration of native African herbs and plants for medicinal use has long been the subject of extensive research. In addition, there has been a time-consuming search for new drugs through molecular pharming [188]. 

Interest in natural products is gaining attention as an integrative approach to the management of sickle cell disease, particularly in Africa, where there is rich biodiversity. As many as 5000 local medicinal foods [189] directly sourced from the wild are used by traditional healers, who serve approximately 80% of the African population [190,191].

Traditional remedies, often based on empirical observations that have proven their accuracy over time, are more popular than pharmaceutical drugs. However, this knowledge is at risk of being lost to younger generations because it is verbally transferred from generation to generation, and they are not eager to inherit this heritage [192]. The bioactive compounds found in these tropical plants interact with the gut microbiota and the available phytonutrients, playing a critical regulatory role in human health [193]. 

Several structured surveys have been conducted in Sub-Saharan Africa, where approximately 80% of the world’s SCA cases occur. These surveys were designed to assess various challenges and dietary aspects of patients with SCA. Their aim was to identify knowledge gaps and prioritize future areas of research [32,35,166,194].

**Figure 3:**
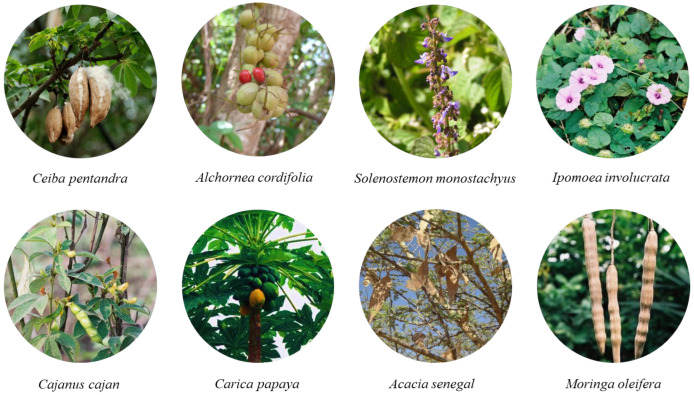
Some of the tropical plants used in SCA in Sub-Saharan Africa.

The potential benefits of nutrition in African SCD patients have been studied primarily in Nigeria [195], with less research in other Sub-Saharan countries, such as Ghana, Sudan, Kenya, Malawi, Tanzania, Cameroon, Ivory Coast, Gabon, and Mali. These studies have explored the use of indigenous medicinal sources, including seed oils from *Solenostemon monostachyus*, *Ipomoea involucrate*, and *Carica papaya* plants, commercial *Cajanus cajan* plant extracts, and *Acacia Senegal* seed oil [166] (Figure 3).

The leaves, root bark, and seeds of *Alchornea cordifolia* (Christmas bush) and *Ceiba pentandra* are wild harvested and cultivated for their medicinal uses in DR Congo. They are used to produce a beverage known as “blood tonic” that is used by individual suffering from SCA [196].

Found throughout Africa, *Moringa oleifera* is rich in many phytochemicals exhibiting antiurolithiatic properties. It is widely used for a vast number of ailments and might be significant in the management of SCD [197] (Table 3). 

A study conducted in Sudan on *Nigella sativa* (black cumin seed) oil extract, which is known to have calcium antagonist antioxidant properties that are useful in the management of SCA, reported significant anti-sickling activity in vitro [198]. 

### 5.2. Mushroom Nutritional Prospects

The typical herbal remedies used by traditional healers in different parts of Africa and elsewhere for the treatment of SCD were recently evaluated [199,200]. Safe, effective, and inexpensive therapeutic agents are urgently needed in Africa, and SCD management can be considered in two main aspects: medical protection and the management of major complications, both of which can be complemented by nutritional approaches [201]. 

Exogenous food-derived microRNAs, obtained through cross-kingdom modulation, may enter the human host from mushrooms, marine algae, and herbal teas. These microRNAs may serve as alternative tactics for new therapeutic effects, enhancing the existence of microRNA interactions between the diet, host, and gut microbiota [202,203,204].

Several reviews have revealed a solid connection between oxidative stress, inflammation, the immune response, and the pathogenesis of SCD, enhancing the role of the dominant natural immune system [205,206].

Despite the use of medicinal plants for millennia, information on African mushrooms in healthcare is less abundant [207,208]. Edible medicinal mushrooms, whether in the form of biomass, extracts, or derivatives, are potential sources of many bioactive products that can regulate immunity [209]. Mushroom bioactive elements exhibit various pharmacological activities, including multitargeting bioactivities, low toxicity, high safety, and affordability, making them valuable medicinal sources [210]. 

These fungal biochemical compounds include carbohydrates (β-glucans/lentinan, trehalose, chitosans) [210], carbohydrate-binding proteins (lectins) [211], mono- and polyunsaturated fatty acids(linoleic, oleic, palmitic) [212], phenolic compounds (caffeic, gallic, cinnamic, p-hydroxybenzoic, p-coumaric, and melatonin) [213], indole compounds (L-trytptophan) [214], vitamins (vitamins B complex, vitamin C, and tocopherols), terpenoids (carotenoids such as β-carotene and lycopene) [215], and unique molecules (e.g. ergothioneine and glutathione) [216,217].

Natural products, which have the ability to affect numerous targets and impact several signaling pathways, are widely recognized for their health benefits. Natural antioxidant-rich foods include berries, avocado, apples, cruciferous vegetables, nuts, olive oil, pulses, tomatoes, and mushrooms [218]. Mushrooms also possess strong anti-inflammatory effects. Despite being underappreciated as a medicinal source in Western countries, there is a growing need for further investigation, including their potential role in complementary prophylactic dietary treatments for sickle cell disease. 

The consumption of edible mushrooms, either unprocessed or as supplements (extracts or as biomass), has been well-documented for many years as powerful instrument in maintaining health, longevity, and quality of life [219]. Their mode of action has been studied and can be attributed to their unique composition, including the essential amino acid (ergothioneine) [220], a type of β-glucans [221,222], specific enzymes, and secondary metabolites [223,224]. The way in which mushroom bioactive components interact with gut microbiota, influencing metabolism and various health disorders, has been the subject of many recent reviews [225,226].

Certain mushrooms are very rich in superoxide-dismutase, glutathione-peroxidase, catalase, and proteases, which are known to interact with transcription factors (e.g., nuclear-factor erythroid 2-related factor 2), thus preserving redox homeostasis in the cell and counteracting oxidative stress [227]. 

The regulatory effects of mushroom active ingredients (e.g., polyphenols) on ferroptosis has been described [228]. Edible mushrooms contain the ferroptosis inhibitor gallic acid, a natural hydroxybenzoic acid, which is also present in various foods such as nuts, red fruits, olive oil, green tea, and vegetables [229]. Ferroptocide is an inhibitor of thioredoxin, a central redox system in mammalian cells, that induces ferroptosis, which is the unique form of programmed death distinct from apoptosis, and maintains redox balances in sickle red blood cells [230,231,232].

Substantial oxidative stress is a prominent contributor to SCD due to a disproportionate yield of reactive oxygen species compared with the ability of antioxidant agents, including enzymes such as SODs, catalase, and glutathione peroxidase, to counteract them. Multiple inflammatory pathways are stimulated in a chain reaction involving sickle cell redox balance, hemolysis, vasculopathy, and regulation in the immune response [205].

Research on *Ganoderma lucidum* mushroom extracts revealed a significant decrease in hemoglobin polymerization rate, enhancing the binding of oxygen, thus preserving or stabilizing the structure of hemoglobin [233]. 

The mushroom *Auricularia auricular*, which is known for its medicinal properties [234] and anti-sickling potential, was recently studied in Nigeria. The study showed that this functional food, which has strong free radical scavenging activity, may reverse and stabilize erythrocyte membrane integrity and morphology. This means that this mushroom may offer a valuable natural option for the treatment and management of sickle cell anemia [235]. 

The mushroom *Hericium erinaceus*, mainly through its polysaccharide components, has traditionally and historically been used as a natural remedy for gastrointestinal disorders and epigastric pain. However, to the best of our knowledge, it has not been tested for use in treating SCD chronic pain [236]. *H. erinaceus* can regulate heat shock proteins (HSP70), which has the potential to offer beneficial health effects [237]. 

Termite mushrooms (*Termitomyces*), a species from Africa and Asia, is used to increase hemoglobin levels (12.2 g/dL) and white blood cells (26,300 cells/mm^3^) [238] and contributes widely to modern medical research because its mycelial biomass expresses strong antioxidant potential [239] (Table 4).

## 6. Concluding Remarks

The phenomenal success of the new genetic treatment for SCD is an important milestone, but it is not yet available to all patients. 

This review assesses the present evidence on the singular mechanisms of traditional and new treatments for SCD, a condition that commonly leads to a dysfunctional immune response. It also offers some suggestions regarding nutritional strategies for SCD patients, particularly those in Sub-Saharan Africa. There is an unprecedented need for clinical research to better explore the potential benefits of nutrition-related interventions involving commonly used medicinal plants. 

There is a global need to identify new ways to maximize the well-being of individuals with SCD, and nutritional interventions could possibly play a pivotal role. It is important to promote the analysis, investigation, and development of functional foods that can provide readily available, cost-effective, and complementary or alternative molecules to decrease the number of crises in individuals with SCD. Hence, the overall nutrition of children with SCD should be improved, leading to an enhanced quality of life. 

A systematic assessment of the drivers of the nutritional status of SCD patients should be a priority for future research. This assessment should also explore the use of various indigenous plant resources in Sub-Saharan Africa in this domain.

## Figures and Tables

**Table 1:** Therapeutic interventions in sickle cell disease.

Therapeutic Intervention	Outcome
Blood transfusion	Reduce the burden of sickled cells [94,95]
Hydroxyurea	Increase fetal hemoglobin (HbF) to stop polymers forming in the sickle hemoglobin [96]
Hematopoietic stem cell transplantation	Reverse the sickle phenotype [97,98]
L-glutamine	Antioxidant effects [99]
Hemoglobin S (HbS) polymerization inhibitors	Prevent HbS polymerization [100]
Monoclonal antibody (crizanlizumab)	Reduce selectin-mediated adhesion [101]
Gene editing therapy (Casgevy™)	Editing faulty gene in a patient’s bone marrow stem cells [102]

**Table 2:** Nutritional and gut microbiome interventions in sickle cell disease.

Nutritional Intervention	Outcome
Microbiota modulation	Reverse established microbial dysbiosis [127,128,129]
Omega-3 fatty acids	Improve VOC rate, markers of inflammation, adhesion, and hemolysis [146]

**Table 3:** Traditional medicine in sickle cell disease.

Plant	
*Alchornea cordifolia*	Traditional use as “blood tonic” [193]
*Ceiba pentandra*	Traditional use as “blood tonic” [193]
*Moringa oleifera*	Antiurolithiatic properties [195]
*Nigella sativa*	Antioxidant properties [196]

**Table 4:** Mushrooms in sickle cell disease.

Mushroom	Outcome
*Ganoderma lucidum*	Decrease in hemoglobin polymerization rate [233]
*Auricularia auricular*	Free radical scavenging activity [235]
*Hericium erinaceus*	Regulation of heat shock proteins (HSP70) [237]
*Termitomyces*	Increase hemoglobin levels and white blood cells [238]
